# Clinical Characterization of the Frequent Exacerbator Phenotype in Asthma

**DOI:** 10.3390/jcm9072226

**Published:** 2020-07-14

**Authors:** Andrea Elio Sprio, Vitina Carriero, Stefano Levra, Carlotta Botto, Francesca Bertolini, Antonino Di Stefano, Mauro Maniscalco, Giorgio Ciprandi, Fabio Luigi Massimo Ricciardolo

**Affiliations:** 1 Department of Clinical and Biological Sciences, University of Turin, San Luigi Gonzaga University Hospital, Orbassano, 10043 Turin, Italy; andrea.sprio@unito.it (A.E.S.); vitina.carriero@unito.it (V.C.); stefano.levra@unito.it (S.L.); carlotta.botto88@gmail.com (C.B.); francesca.bertolini@unito.it (F.B.); 2 Department of Pneumology and Laboratory of Cytoimmunopathology of the Heart and Lung, Istituti Clinici Scientifici Maugeri SpA, IRCCS, Veruno, 28010 Novara, Italy; antonino.distefano@icsmaugeri.it; 3 Pulmonary Rehabilitation Unit, Istituti Clinici Scientifici Maugeri SpA, IRCCS, Telese Terme, 82037 Benevento, Italy; mauromaniscalco@hotmail.com; 4 Allergy Clinic, Casa di Cura Villa Montallegro, 16145 Genoa, Italy; gio.cip@libero.it

**Keywords:** asthma, airway obstruction, nasal polyps, nitric oxide, IgE

## Abstract

Background: Asthma exacerbation is episodic worsening of respiratory symptoms in conjunction with the deterioration of lung function, which may occur independently from the asthma severity hampering asthmatics’ quality of life. This study aimed to characterize the patient phenotype more prone to asthma exacerbation (oral corticosteroid burst ≥2 per year) to allow the proper identification of such patients. Methods: This real-life, observational, cross-sectional study evaluated 464 asthmatic patients stratified according to the asthma exacerbations experienced in the previous year. Clinical, functional, and blood parameters were retrieved from chart data and were representative of patients in stable conditions. Results: The frequent asthma exacerbator was more commonly female, suffered from chronic rhinosinusitis with nasal polyposis, had reduced lung function and peripheral oxygen saturation, and had increased daily activity limitations. These patients often had severe asthma and more frequently needed hospitalization in their lives. Furthermore, the frequent asthma exacerbator had higher concentrations of serum immunoglobulin E (IgE) and exhaled nitric oxide with cut-off risk values of 107.5 kU/L (OR = 4.1) and 43.35 ppb (OR = 3.8), respectively. Conclusions: This study illustrates the clinical features of the frequent asthma exacerbator phenotype. Nevertheless, serum IgE and exhaled nitric oxide could allow the identification of this phenotype and the establishment of an appropriate therapeutic approach.

## 1. Introduction

Asthma is a heterogeneous disorder characterized by chronic complex inflammatory status and reversible airflow obstruction. This respiratory condition displays episodic worsening of respiratory symptoms in conjunction with a deterioration of lung function (so-called asthma exacerbation (AE)), which requires changes in treatment to reacquire asthma control [1]. The AE is defined as severe when it necessitates the use of systemic corticosteroids for at least three days, whereas it is defined as serious when emergency department/hospital admission occurs and the condition eventually requires mechanical ventilation [1,2]. The latter is considered a life-threatening event, representing the leading cause of morbidity and mortality related to asthma. In this context, the occurrence of AE reduces the patient’s quality of life significantly, inducing a state of stress, anxiety, and fear [3].

Furthermore, AE has a substantial economic impact on the healthcare system, as it is responsible for a considerable portion of the annual economic burden correlated with asthma [3]. A previous report estimated that the uncontrolled asthma patient costs about four times more than a well-controlled one [4]. Therefore, understanding the factors related to the development of exacerbations must be considered of great socioeconomic importance.

AE is a common feature of patients affected by severe asthma [5], a condition that requires a high dose of inhaled corticosteroids plus a second controller (and/or systemic corticosteroids) [2,6]. Thereby, the literature describes different aspects and characteristics of these patients. Phenotype and endotype classifications have been provided based on the involved inflammatory response, immune mediators, or molecular mechanisms [7,8].

The frequency of AE is positively correlated to high counts in sputum and blood eosinophils [9,10,11,12], and therapies against type-2 immune response mediators showed efficacy in reducing the AE rate [13]. However, discrepancies in these correlations suggest that the exacerbation rate could not be strictly associated with systemic type-2 inflammation [14]. Indeed, higher neutrophil counts in both sputum and blood of asthmatic patients with exacerbations are reported in the literature [15,16]. A dual immune response mediated by both type-2 and non-type-2 cytokines has been proposed [17] and linked with exacerbation in the severe asthmatic patient [18].

Nevertheless, AE can be experienced irrespectively of asthma severity [19]. In this context, the analysis of phenotypes based on exacerbation frequency is required. Thus far, the literature has lacked a systematic study on the risk of developing AE. We analyzed a large cohort of patients affected by mild to severe asthma in a real-world setting and stratified them according to the AE frequency, seeking to describe the main features and risks correlated to the frequent exacerbator phenotype.

## 2. Methods

### 2.1. Patients

This real-life, observational, cross-sectional study, which ended in September 2019, evaluated chart data from 464 asthmatic patients who were referred to the “Chronic Obstructive Pulmonary Disease and Severe Asthma” Service at the San Luigi Gonzaga University Hospital (Orbassano, Torino, Italy).

The inclusion criterion was the confirmed diagnosis of mild to severe asthma according to the Global Initiative for Asthma (GINA) and ERS/ATS guidelines [2,20]. All analyses were only performed on patients without AE symptoms and in stable condition. All the patients were optimally treated. On each visit, self-reported adherence to inhaled therapy (including inhaled corticosteroids (ICS)) was questioned, and the inhalation technique was checked. The prescribed ICS dose was adjusted for the percentage of self-reported therapy adherence and then recorded. In this study, patients with poor adherence (<50%) or with poor inhalation technique were excluded because of the low reliability of their data and to ensure the evaluation of only optimally treated patients.

This study was performed following the Declaration of Helsinki and approved by the San Luigi Gonzaga University Hospital Review Board (Prot. No. 4478/2017). All participants provided written informed consent.

### 2.2. Study Design and Setting

Patients were stratified based on their severe AE frequency in the year (AE/y) preceding the last visit. We considered episodes of severe AE to be those requiring systemic corticosteroid (CS) bursts for more than three consecutive days [2]. In a first instance, we used the cut-off of 2 AE/y; a patient with <2 AE/y was classified as a nonfrequent exacerbator (nFE; *n* = 367), whereas a patient who experienced ≥2 AE/y was classified as frequent exacerbator (FE; *n* = 97) [11,21]. Then, we further subdivided these categories into non- (NE; AE/y = 0, *n* = 248), occasional (OE; AE/y = 1, *n* = 119), mildly frequent (mFE; AE/y = 2, *n* = 43), and highly frequent (hFE; AE/y ≥ 3, *n* = 54) exacerbator groups of patients.

Life-span history of serious (emergency room access and hospitalization) [2] and near-fatal (tracheal intubation and mechanical ventilation) AE was also retrieved from chart data and reported.

### 2.3. Evaluation of Clinical, Functional, and Blood Parameters

Collected data included demographic parameters (age, gender, BMI, and smoking history (using ≥10 pack-years as a cut-off)), clinical history with the diagnosis of comorbidities, pulmonary function tests, fractional exhaled nitric oxide (FeNO), and asthma control test (ACT) questionnaire [22,23].

We evaluated pulmonary function parameters assessing spirometry and lung volumes before and 15 min after albuterol administration (400 µg) using body plethysmograph (Vmax Encore 62, Carefusion, Germany). FeNO was quantified using the FeNO+ instrument (Medisoft, Sorinnes, Belgium) according to the manufacturer’s instruction. Chronic rhinosinusitis with (CRSwNP) or without nasal polyps (CRSsNP) and aspirin-exacerbated respiratory disease (AERD) were diagnosed according to the EPOS-2020 guideline [24].

Skin prick tests, serum total IgE levels, and consistent history identified allergic patients according to validated criteria [25]. Patients sensitized to ≥2 allergens have been considered as polysensitized [26].

Blood neutrophil and eosinophil counts were performed using a Cell-Dyn Sapphire (Abbott, Rome, Italy) in routinely performed hematological examinations. 

Asthma control was evaluated through the ACT questionnaire [22,23], whereas the asthma severity was classified based on the treatment strategy coded by GINA guidelines (box 7: GINA treatment steps) [20]. In particular, we referred to treatments in GINA treatment steps 1–2 as mild asthma, GINA treatment steps 3–4 as moderate asthma, and GINA treatment step 5 as severe asthma. The first item of the ACT questionnaire was used to evaluate daily activity limitations. 

### 2.4. Statistical Analysis

The ROUT method was employed to detect and exclude outliers [27]. Data distributions were assessed by the D’Agostino–Pearson test to detect departure from normality. Differences between nFE and FE groups were compared using the unpaired t-test or the Mann–Whitney test. Differences between the four different exacerbation groups were compared with one-way ANOVA (with Tukey post hoc test) or Kruskal–Wallis test (with Dunn’s multiple comparisons test). Chi-squared (χ^2^) tests were used to compare frequencies. Binary logistic regression (LR) evaluated independent risk factors for the occurrence of severe AE. Results were considered statistically significant when *p* < 0.05. Receiver operating characteristic (ROC) curves were used to determine optimal cut-off values of possible predictors of highly frequent exacerbation; area under the ROC curve (AUC) measured the accuracy of each score. Odds ratio (OR) was used to estimate the odds of becoming a frequent exacerbator. LR was performed with IBM SPSS Statistics 24 (IBM Corp., Armonk, NY, USA), whereas the other statistical evaluations were performed with GraphPad Prism 8.2.1 (GraphPad Software, San Diego, CA, USA).

## 3. Results

### 3.1. Clinical Characteristics and Comorbidities

The distribution of patients among groups was homogeneous in terms of both age and BMI (Table 1). Similarly, no differences in terms of smoking history were detected between nFE and FE patients; although a few patients in the hFE group were former smokers, none of them were current smokers. Women were more frequently FE patients (*p* < 0.05) and particularly more frequently mFE patients (*p* < 0.01 and *p* < 0.05 vs. NE and OE, respectively).

The distribution of allergic patients in the groups was homogeneous. Likewise, no differences were detected considering the sensitization to perennial or seasonal antigens. 

FE patients suffered more frequently from severe asthma than the nFE patients (OR 4.3, 95%CI 2.6–7.1, *p* < 0.001) and were more prone to be hospitalized due to asthma exacerbations (serious AE, OR 2.7, 95%CI 1.6–4.5, *p* < 0.001). Within the FE group, hFE patients were more likely to have a history of AE-related hospitalization than all the others (OR 2.8, 95%CI 1.5–5.2, *p* < 0.01).

Prevalence of the main comorbidities was similar among studied groups, except for nasal polyposis (CRSwNP + CRSwNP-AERD, Table 2), which were more common in the FE patients (*p* < 0.05). In particular, hFE patients suffered more frequently from nasal polyposis when compared to NE (*p* < 0.01), OE (*p* < 0.001), and mFE (*p* < 0.05) patients. FE had an OR = 2.0 (95%CI 1.2–3.4, *p* < 0.05), while hFE patients had an OR = 3.2 (95%CI 1.7–5.9, *p* < 0.001) to develop nasal polyps. Moreover, hFE patients had more CRSwNP than NE and OE patients (*p* < 0.05) and more CRSwNP-AERD than OE patients (*p* < 0.05). In this context, hFE patients had an OR = 2.5 (95%CI 1.2–5.0, *p* < 0.05) and an OR = 3.5 (95%CI 1.3–9.0, *p* < 0.05) to suffer CRSwNP and CRSwNP-AERD, respectively.

### 3.2. Functional Parameters

**Figure 1:**
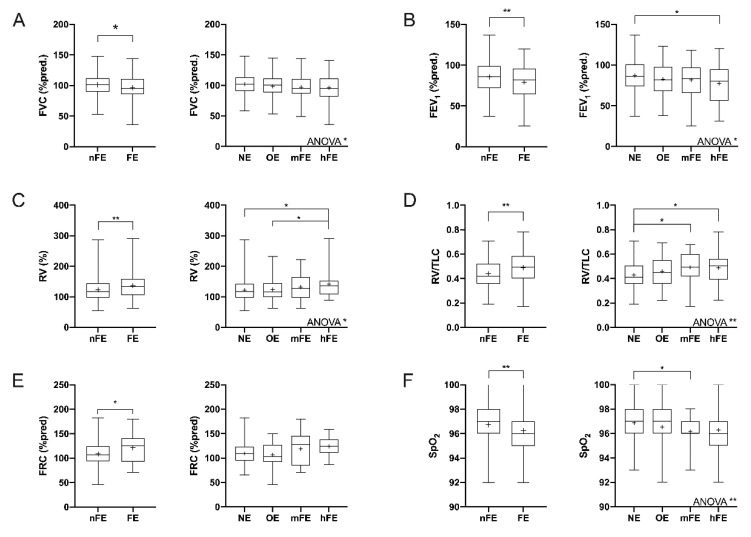
Functional parameters in the asthmatic population after stratification according to the number of exacerbations. (**A**) FVC %pred; (**B**) FEV_1_ %pred; (**C**) RV % (%pred); (**D**) RV/TLC ratio; (**E**) FRC %pred; (**F**) blood oxygen saturation. Box plots represent data from the first to the third quartile; whiskers represent the minimum and maximum values; “^+^” indicates the mean value. nFE: nonfrequent exacerbator; FE: frequent exacerbator; NE: non-exacerbator; OE: occasional exacerbator; mFE: mildly frequent exacerbator; hFE: highly frequent exacerbator; ANOVA: analysis of variance. * *p* < 0.05; ** *p* < 0.01.

The occurrence of asthma exacerbations induced pulmonary function abnormalities. FVC %pred and FEV_1_ %pred were reduced in the FE group compared to the nFE group (*p* < 0.05 and *p* < 0.01, respectively; Figure 1A,B). These defective functional values were proportional to the increased number of severe AE experiences (ANOVA, *p* < 0.05). Furthermore, a significant difference in terms of FEV_1_ %pred was detected between NE and hFE patients (*p* < 0.05). 

RV %pred, RV/TLC ratio, and FRC %pred were increased (*p* < 0.01, *p* < 0.01, and *p* < 0.05, respectively) in FE patients in comparison to nFE patients (Figure 1C–E). Analysis of variance detected significant increments in RV %pred (*p* < 0.05) and RV/TLC ratio (*p* < 0.01) but only a trend (*p* = 0.064) in regard to FRC %pred. RV was higher in hFE patients than in those belonging to NE and OE groups (both *p* < 0.05), while the RV/TLC was greater in both mFE and hFE groups than in the NE group (both *p* < 0.05).

No differences in terms of FEV_1_/FVC ratio (%) and TLC %pred were detected among evaluated groups, nor were differences detected concerning volume and flow changes (mL) of FVC and FEV_1_ after bronchodilation (Table S1). 

Although no differences were detected considering the heart rate of patients (Table S1), blood oxygen saturation levels (SpO_2_) decreased as the number of AE/y increased (Figure 1F). FE patients had lower SpO_2_ than nFE ones (*p* < 0.01), and there was a progressive SpO_2_ reduction from NE to hFE patients (ANOVA, *p* < 0.01). 

### 3.3. Asthma Control

**Figure 2:**
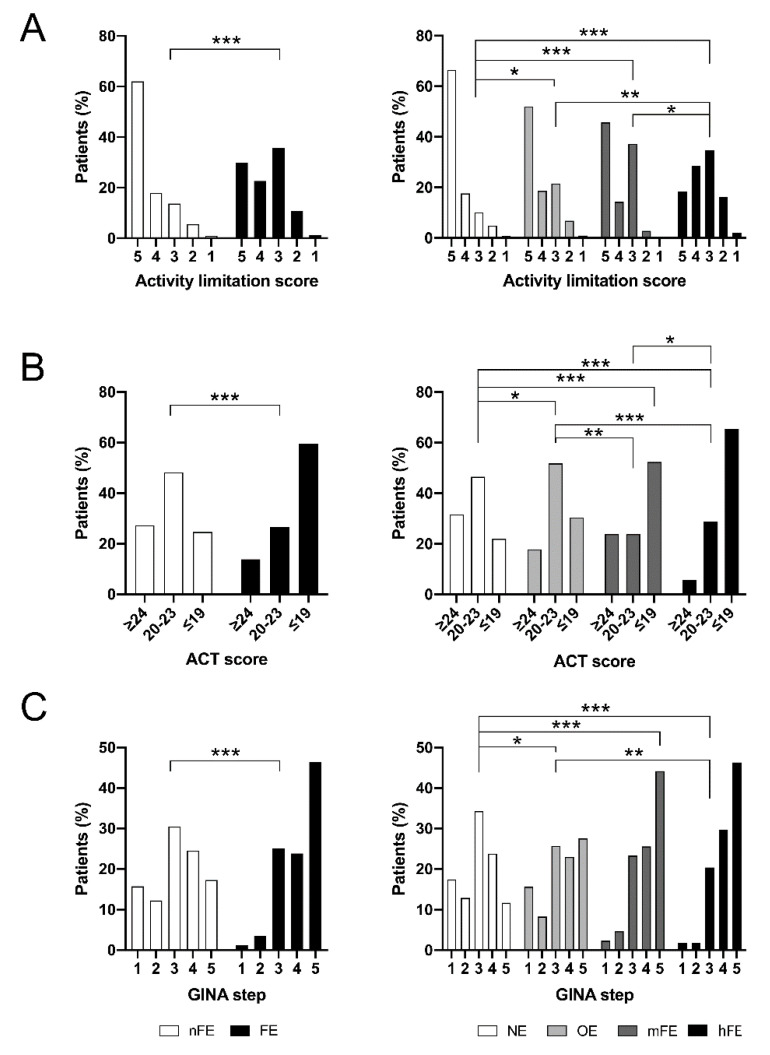
Asthma control in the asthmatic population after stratification according to the number of exacerbations. Results of (**A**) limitations to daily activity and (**B**) asthma control test (ACT) improve as scores increase. (**C**) The resulting treatment step according to Global Initiative for Asthma (GINA) improves as score decreases. * *p* < 0.05; ** *p* < 0.01; *** *p* < 0.001.

Daily activity limitations (DALs) were more common in FE patients than nFE patients (*p* < 0.001; Figure 2A). Limitation occurrences were proportional to the AE/y: hFE patients had more impairments than the others (*p* < 0.05, *p* < 0.01, and *p* < 0.001 versus mFE, OE, and NE, respectively), whereas activities were more limited in mFE and OE patients than in NE patients (*p* < 0.001 and *p* < 0.05, respectively). No differences were detected between mFE and OE patients. Considering a DAL score ≤3 as a threshold, we detected that FE patients were more prone (41.2%) to develop limitations than the nFE ones (18.0%, OR = 3.2, 95%CI 1.9–5.2, *p* < 0.001). Furthermore, hFE patients suffered from a worse situation (48.2%) than the others (19.5%, OR = 3.8, 95%CI 2.1–6.9, *p* < 0.001).

FE patients suffered more frequently from uncontrolled asthma than nFE patients (*p* < 0.001; Figure 2B). Results of ACT worsened with the increase of AE/y (*p* < 0.001). Well-controlled asthma was more frequent in NE, while few cases were present in the hFE group; the OE and mFE groups had a comparable percentage of well-controlled patients. Partially controlled asthma was common in the NE and OE groups. In contrast, the percentage of patients who had uncontrolled asthma increased progressively from the NE to the hFE group, in which the percentage was at its maximum.

### 3.4. Asthma Treatment

FE patients were classified predominantly as belonging to the GINA treatment step 5 and, to a lesser extent, were also classified as steps 3 and 4. Otherwise, nFE patients were mainly classified as step 3 (*p* < 0.001; Figure 2C) [20]. Higher GINA treatment step classifications were characteristic of hFE and mFE groups. Among them, the hFE group showed higher GINA treatment step classifications than the OE group (*p* < 0.01), whereas NE patients showed a milder classification than the others (*p* < 0.01 vs. OE and *p* < 0.001 vs. mFE and hFE).

FE patients received higher doses of ICS than nFE patients (*p* < 0.001; Table 3). Treatment of hFE and mFE patients required ICS doses higher than those administered to OE patients (*p* < 0.001 and *p* < 0.05, respectively). The lowest doses were given to NE patients (*p* < 0.001 vs. hFE and mFE and *p* < 0.01 vs. OE). Moreover, hFE patients underwent OCS treatment more frequently than those of the other groups (*p* < 0.05 vs. mFE, *p* = 0.075 vs. OE, and *p* < 0.001 vs. NE). FE patients used long-acting β_2_ agonists (LABA) and/or long acting-muscarinic antagonists (LAMA) more frequently (*p* < 0.001 and *p* < 0.05, respectively) than nFE patients. The hFE group used more LABA than the OE (*p* < 0.01) and NE (*p* < 0.001) groups and used more LAMA than the NE group (*p* < 0.05), whereas mFE patients used more LABA than NE patients (*p* < 0.01). Differences in biological drug administration were detected only for mepolizumab, which was given more frequently to FE (*p* < 0.001) and, in particular, hFE patients than those belonging to OE (*p* < 0.01) or NE (*p* < 0.001) groups.

### 3.5. T2-High Biomarkers

**Figure 3:**
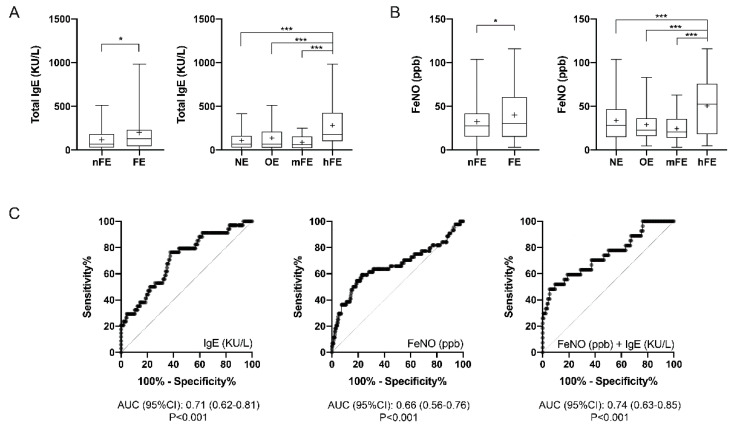
T2 biomarkers in the asthmatic population after stratification according to the number of exacerbations: (**A**) serum IgE concentrations, (**B**) FeNO values, and (**C**) their respective and cumulative ROC curve analyses. Box plots represent data from the first to the third quartile; whiskers represent the minimum and maximum values; “ ^+^ ” indicates the mean value. * *p* < 0.05; *** *p* < 0.001.

FE patients had higher levels of white blood cells (WBC) (*p* < 0.05), but no differences in terms of blood neutrophil or blood eosinophil counts were detected among the analyzed groups (Table 1, Figure S1). Nevertheless, levels of IgE in the blood of FE patients were higher than those in nFE patients (*p* < 0.05), as the hFE group had significantly higher levels than the other groups (*p* < 0.001; Figure 3A). Similarly, FeNO was higher in FE (*p* < 0.05) patients than in nFE patients due to the higher concentrations detected in hFE patients (*p* < 0.001 against all the other groups; Figure 3B).

Thus, the hFE group was compared to all the other categories combined, and ROC curve analyses allowed the detection of possible cut-off values (Figure 3C). Patients with serum total IgE concentration >107.5 kU/L had almost 4 times the odds to belong to the hFE group (OR 4.1 (95%CI 1.7–8.8); *p* < 0.001); sensitivity was 93.6%, specificity 21.6%, PPV 54.6%, and NPV 77.1%. Furthermore, patients with FeNO levels >43.35 ppb had more than 3-fold the probability to be hFE patients (OR 3.8 (95%CI 2.0–7.1); *p* < 0.001); sensitivity was 93.1%, specificity 21.8%, PPV 72.2%, and NPV 59.1%. ROC curve analyses of the cumulative probability obtained by combining serum IgE and FeNO concentrations through the LR formula

(1) $$P=\frac{1}{1+e^{-[-3.819+\left(0.026\times FeNO\;ppb\right)+\left(0.005\times IgE\;kU/L\right)}},$$

allowed us to detect that patients with value of *P* >0.2522 were about 16 times more likely to be hFE patients (OR 16.5 [95%CI 5.9–45.4]; *p* < 0.001); sensitivity was 92.0%, specificity 59.1%, PPV 97.7%, and NPV 48.2%.

## 4. Discussion

In this study, we analyzed the characteristics of 464 patients suffering from mild to severe asthma. Based on AE/y, we classified patients as either nonfrequent (nFE) or frequent exacerbator (FE) patients, further subdividing them into non- (NE), occasional (OE), mildly frequent (mFE), and highly frequent exacerbator (hFE) groups. Clinically, we detected that FE patients are often women and suffered more frequently from sinusitis and nasal polyposis. Similarly, pulmonary function and SpO_2_ were hampered in FE patients, with consequent daily activity limitations. Exacerbating patients had a higher probability of suffering from severe asthma and had a history of serious AE. FE patients also had poor asthma control. Therefore, their treatment included ICS (with or without LAMA and LABA) at higher doses than those administered to nFE patients and frequently included OCS or mepolizumab. The therapeutic strategy (GINA treatment step) varied according to the shift from NE to hFE. FE patients had a higher count of WBC than their more controlled counterparts, although levels of neutrophils and eosinophils were comparable between the two populations. Interestingly, hFE patients had higher levels of serum IgE and FeNO than the other groups. Using ROC curves, we detected possible cut-off values able, alone or in combination, to predict the probability of belonging to the hFE group.

Although BMI and age have been described as factors having a role in AE occurrence, they did not appear to be involved in our cohort. This discrepancy could be mainly due to the mean age (57.7 to 60.4 years) and BMI (25.7 and 27.2) within our groups, which were higher than risk thresholds reported in the literature (age > 45 years and BMI ≈ 23.5) [28,29]. Gender differences are otherwise present and in accord with the literature: patients affected by frequent exacerbation are often women, who generally are more susceptible because of hormone-mediated mechanisms [30,31]. However, after a further stratification according to the number of AE/y, the gender ratio of hFE patients became similar to those of patients belonging to the nFE groups (NE and OE). The unbalance detected in the mFE group is not fully understood and could be related to the higher mean age in this population (60.4 years), but further analyses are still required. Finally, cigarette smoking history did not promote the onset of severe AE significantly in our population, and thus it cannot be considered as a risk factor. It should be noted that there were fewer former smokers and no current smokers present in the more exacerbating hFE group. 

As the number of exacerbations increased, progressive deterioration in lung function occurred. In particular, FE patients exhibited greater airway obstruction at both proximal (airflow limitation as determined by reduced FEV_1_) and peripheral levels (air trapping as determined by lower FVC and higher RV and RV/TLC ratio; lung hyperinflation as determined by increased FRC) than the nonfrequent ones. This condition could be the cause of the reduced SpO_2_ detected in FE patients, confirming the respiratory pathophysiology axiom. Lack of alteration regarding heart rate could be explained by the SpO_2_ remaining, on average, higher than 96%.

The main consequence of lung function deterioration was daily activity limitations, which represent the first clinical sign of respiratory disability. Impairments affected OE and mFE patients, but they had a greater impact on hFE patients. Activity limitation is more common in FE (OR 3.2) than nFE patients, where patients classified as hFE had the highest probability (OR 3.8). In this context, it should be taken into consideration that there is a psychological component that can increase the intensity of the perceived limitation [32].

Asthma severity and control in FE patients were predominantly worsened and thus required high-intensity pharmacological treatment [20]. Patients suffering from numerous AE/y often required therapeutic strategies classifiable in higher GINA treatment steps (above all in step 5). Despite these treatments, FE patients were more prone (OR 2.7) to have a history of serious exacerbation (hospitalization, ICU stay, or mechanical ventilation) [2] than nFE patients. The hFE patient remains that with the highest probability of hospitalization (OR 2.8). 

Mepolizumab treatment was more frequent in FE patients than in nFE patients. However, we detected three NE patients and four OE patients in treatment since 2017. These patients met the mepolizumab eligibility [33] at the beginning of the treatment. Otherwise, no significant changes in terms of omalizumab administration have been detected. This is likely because the frequency of atopy was similar between patient groups, which were comparable in terms of sensitization to perennial and seasonal allergens.

Therapy against IL-5 hampers eosinophil maturation, activation, and survival, whereas the corticosteroid therapy causes eosinophil apoptosis, favoring the switch to a more neutrophilic phenotype of asthma when administered at the highest dose [34]. No changes have been observed in terms of both blood eosinophils and neutrophils between FE and nFE patients, despite the administration of higher corticosteroid doses and mepolizumab in FE patients. Otherwise, a significant increment of WBC in FE patients was evident, possibly due to the subclinical respiratory infection [35] or systemic inflammation [36] that underlies the severe AE occurrence.

On the basis of these findings, the high-intensity pharmacological treatment apparently dissociated eosinophil count from the other T2 biomarkers. The anti-IL-5 treatment reduces eosinophil count but does not affect IgE concentrations and FeNO values [37,38], which are modulated by the IL4/13 signaling [39,40]. Indeed, the predominance of a T2-high inflammatory status is evident above all in the most exacerbating hFE group. These patients were characterized by a higher incidence of nasal polyposis (OR 3.2), a potential eosinophilic-derived disease [41], and higher concentrations of serum total IgE and FeNO than those belonging to the other groups.

Detection of these two biomarkers predicts exacerbation [18,42]; it can be useful to discriminate patients at higher risk of developing frequently severe AE. In particular, we detected that serum IgE concentrations and FeNO values greater than 107.5 kU/L and 43.35 ppb are moderate predictors of belonging to the hFE group (OR 4.1 and 3.8, respectively). Nevertheless, their cumulative probability (when *P* > 0.2522) provides better results and is a more reliable predictor (OR 16.1). We may also infer that FE patients could express a combination of T2 and Th17 immune response in the airways, as previously demonstrated [18,43,44].

## 5. Conclusions

The main limitations of this study are represented by the real-life design and the intrinsic characteristics of our population, consisting mainly of adult and elderly patients. In this point of view, there is a conflict with the previous literature in that differences in age, BMI, and, to some extent, gender may have been underestimated.

In conclusion, we described the characteristics of asthmatic patients prone to various degrees of asthma exacerbation. In particular, we highlight the highly frequent exacerbator as a phenotype with the worst prognosis due to a high probability of developing respiratory disability and a history of serious exacerbations that required hospitalization. Nevertheless, elevated FeNO and IgE levels characterized the hFE phenotype and are likely exploitable for its identification, allowing the appropriate therapeutic approach.

## Figures and Tables

**Table 1:** Descriptive statistics of the asthmatic population.

Demographic and Clinical Parameters	nFE (*n* = 367)	FE (*n* = 97)	NE (*n* = 248)	OE (*n* = 119)	mFE (*n* = 43)	hFE (*n* = 54)
Age (years)	58.2 ± 15.6	59.9 ± 13.9	57.7 ± 15.6	59.2 ± 15.6	60.4 ± 14.1	59.5 ± 13.8
Gender (F)	219/367 (59.7%)	71/97 (73.2%) *	145/248 (58.5%)	74/119 (62.2%)	35/43 (81.4%) ^## §^	36/54 (66.7%)
BMI (kg/m^2^)	27.1 ± 5.8	26.4 ± 4.8	27.2 ± 5.9	26.9 ± 5.5	25.7 ± 4.3	26.9 ± 5.2
Current smokers (≥10 PY)	27/367 (7.3%)	1/97 (1.0%) *	19/248 (7.7%)	8/119 (6.7%)	1/43 (2.3%)	0/54 (0.0%)
Former smokers (≥10 PY)	81/367 (22.1%)	18/97 (18.6%)	55/248 (22.2%)	26/119 (21.8%)	7/43 (16.3)	11/54 (20.4%)
Allergy	193/367 (52.6%)	55/97 (56.7%)	134/248 (54.0%)	59/119 (49.6%)	24/43 (55.8%)	31/54 (57.4%)
Polysensitization	165/193 (85.5%)	43/55 (78.2%)	117/134 (87.3%)	48/59 (81.4%)	19/24 (79.2%)	24/31 (77.4%)
Seasonal allergen sensitization	161/193 (83.4%)	47/55 (85.4%)	114/134 (85.1%)	47/59 (79.7%)	22/24 (91.7%)	25/31 (80.6%)
Perennial allergen sensitization	144/193 (74.6%)	38/55 (69.1%)	102/134 (76.1%)	42/59 (71.2%)	16/24 (66.7%)	22/31 (71.0%)
Mild asthma (GINA steps 1–2)	101/367 (27.5%)	5/97 (5.1%) ***	75/248 (30.2%)	26/119 (21.9%)	3/43 (7.0%) ^##^	2/54 (3.7%) ^### §§^
Moderate asthma (GINA steps 3–4)	207/367 (56.4%)	48/97 (49.5%)	144/248 (58.1%)	63/119 (52.9%)	21/43 (48.8%)	27/54 (50.0%)
Severe asthma (GINA step 5)	59/367 (16.1%)	44/97 (45.4%) ***	29/248 (11.7%)	30/119 (25.2%) ^##^	19/43 (44.2%) ^### §^	25/54 (46.3%) ^### §^
Age at asthma onset	37.5 ± 19.3	36.5 ± 17.1	37.7 ± 19.5	37.2 ± 19.0	37.2 ± 16.8	35.9 ± 17.5
Asthma onset (<18 years)	76/367 (20.7%)	21/97 (21.6%)	54/248 (21.8%)	22/119 (18.5%)	9/43 (20.9%)	12/54 (22.2%)
Asthma duration (years)	22.2 ± 17.0	24.9 ± 16.5	21.0 ± 16.4	22.2 ± 16.6	23.5 ± 13.2	24.4 ± 16.9
Serious asthma exacerbation history	54/367 (14.7%)	31/97 (32.0%) ***	30/248 (12.1%)	24/119 (20.2%)	12/43 (27.9%) ^#^	19/54 (35.2%) ^###^
Near-fatal asthma exacerbation history	3/54 (5.6%)	2/31 (6.4%)	0/30 (0.0%)	3/24 (12.5%)	1/12 (8.3%)	1/19 (5.3%)
T2-high phenotype	293/366 (80.0%)	84/97 (86.6%)	203/247 (82.2%)	90/119 (75.6%)	35/43 (81.4%)	49/54 (90.7%)
White blood cells (cells/mm^2^)	7233 ± 2091	7565 ± 1770 *	7149 ± 1816	7412 ± 2580	7286 ± 1642	7788 ± 1851
Blood eosinophils (cells/mm^2^)	311.4 ± 239.5	325.8 ± 282.5	315.4 ± 245.5	302.8 ± 226.7	290.5 ± 239.5	353.9 ± 312.1
Blood neutrophils (cells/mm^2^)	4239 ± 1802	4252 ± 1390	4116 ± 1446	4486 ± 2362	4100 ± 1144	4368 ± 1556

Serious asthma exacerbation: emergency room access and hospitalization; near-fatal asthma exacerbation: tracheal intubation and mechanical ventilation; PY: pack-year; GINA: Global Initiative for Asthma; T2-high: presence of allergy, blood eosinophils >300 cell/µL, serum IgE >100 IU/mL, or FeNO >30 ppb; * *p* < 0.05, *** *p* < 0.001 vs. nFE; ^#^ *p* < 0.05, ^##^ *p* < 0.01, ^###^ *p* < 0.001 vs. NE; ^§^ *p* < 0.05, ^§§^ *p* < 0.01.

**Table 2:** Prevalence of comorbidities in the asthmatic population.

Comorbidities	nFE (*n* = 367)	FE (*n* = 97)	NE (*n* = 248)	OE (*n* = 119)	mFE (*n* = 43)	hFE (*n* = 54)
Rhinitis	246/367 (67.0%)	69/97 (71.1%)	173/248 (69.8%)	73/119 (61.3%)	31/43 (72.1%)	38/54 (70.4%)
CRSsNP	67/367 (18.2%)	19/97 (19.6%)	43/248 (17.3%)	24/119 (20.2%)	11/43 (25.6%)	8/54 (14.8%)
CRSwNP	41/367 (11.2%)	18/97 (18.6%)	30/248 (12.1%)	11/119 (9.2%)	5/43 (11.6%)	13/54 (24.1%) ^#§^
CRSwNP-AERD	13/367 (3.5%)	7/97 (7.2%)	11/248 (4.4%)	2/119 (1.7%)	1/43 (2.3%)	6/54 (11.1%) ^§^
CRSwNP + CRSwNP-AERD	54/367 (14.7%)	25/97 (25.8%) *	41/248 (16.5%)	13/119 (10.9%)	6/43 (14.0%)	19/54 (35.2%) ^## §§§ †^
Bronchiectasis	23/367 (6.3%)	11/97 (11.3%)	15/248 (6.0%)	8/119 (6.7%)	5/43 (11.6%)	6/54 (11.1%)
Pneumonia history	39/367 (10.6%)	13/97 (13.4%)	33/248 (13.3%)	6/119 (5.0%)	6/43 (14.0%)	7/54 (13.0%)
Obstructive sleep apnea syndrome	20/367 (5.4%)	4/97 (4.1%)	14/248 (5.6%)	6/119 (5.0%)	2/43 (4.6%)	2/54 (3.7%)
Gastroesophageal reflux disease	85/367 (23.2%)	26/97 (26.8%)	62/248 (25.0%)	23/119 (19.3%)	13/43 (30.2%)	13/54 (24.1%)
Obesity	89/367 (24.2%)	21/97 (21.6%)	57/248 (23.0%)	32/119 (26.9%)	6/43 (14.0%)	15/54 (27.8%)
Diabetes	23/367 (6.3%)	7/97 (7.2%)	13/248 (5.2%)	10/119 (8.4%)	2/43 (4.6%)	5/54 (9.3%)
Hypertension	119/367 (32.4%)	30/97 (30.9%)	85/248 (34.3%)	34/119 (28.6%)	14/43 (32.6%)	16/54 (29.6%)
Heart failure	6/367 (1.6%)	2/97 (2.1%)	5/248 (2.0%)	1/119 (0.8%)	1/43 (2.3%)	1/53 (1.8%)
Acute myocardial infarction	20/367 (5.4%)	3/97 (3.1%)	11/248 (4.4%)	9/119 (7.6%)	3/43 (7.0%)	0/54 (0.0%)
Arrhythmia	27/367 (7.4%)	7/97 (7.2%)	17/248 (6.8%)	10/119 (8.4%)	3/43 (7.0%)	4/54 (7.4%)
Anxiety depression syndrome	52/367 (14.2%)	14/97 (14.4%)	35/248 (14.1%)	17/119 (14.3%)	8/43 (18.6%)	6/54 (11.1%)
Osteoporosis	31/397 (8.4%)	6/97 (6.2%)	20/248 (8.1%)	11/119 (9.2%)	2/43 (4.6%)	4/54 (7.4%)
Arthropathy	31/367 (8.4%)	9/97 (9.3%)	18/248 (7.3%)	13/119 (10.9%)	2/43 (4.6%)	7/54 (13.0%)
Chronic pain	23/367 (5.9%)	7/97 (7.2%)	13/248 (5.2%)	10/119 (8.4%)	3/43 (7.0%)	4/54 (7.4%)

CRSsNP: chronic rhinosinusitis without nasal polyposis; CRSwNP: chronic rhinosinusitis with nasal polyposis; CRSwNP-AERD: chronic rhinosinusitis with nasal polyposis and aspirin-exacerbated respiratory disease; * *p* < 0.05 vs. nFE; ^#^ *p* < 0.05, ^##^ *p* < 0.01 vs. NE; ^§^ *p* < 0.05, ^§§§^ *p* < 0.001 vs. OE; ^†^ *p* < 0.05 vs. mFE.

**Table 3:** Pharmacological treatments in the asthmatic population.

Treatment	nFE (*n* = 367)	FE (*n* = 97)	NE (*n* = 248)	OE (*n* = 119)	mFE (*n* = 43)	hFE (*n* = 54)
ICS	307/367 (83.6%)	95/97 (97.9%) ***	205/248 (82.7%)	102/119 (85.7%)	42/43 (97.7%) ^#^	53/54 (98.1%) ^## §^
ICS/day (μg BDP-HFA)	263.5 ± 201	446.4 ± 237.6 ***	239.1 ± 176	314.3 ± 238.0^##^	423.3 ± 235.9 ^### §^	464.8 ± 239.6 ^### §§§^
LABA	265/397 (72.2%)	92/97 (94.8%) ***	172/248 (69.4%)	93/119 (78.2%)	40/43 (93.0%) ^##^	52/54 (96.3%) ^### §§^
LAMA	56/367 (15.3%)	25/97 (25.8%) *	37/248 (14.9%)	19/119 (16.0%)	9/43 (20.9%)	16/54 (29.6%) ^#^
OCS (≥6 months/year)	9/367 (2.4%)	7/97 (7.2%)	4/248 (1.6%)	5/119 (4.2%)	0/43 (0.0%)	7/54 (13.0%) ^### †^
Omalizumab	18/367 (4.9%)	9/88 (9.3%)	12/248 (4.8%)	6/119 (5.0%)	6/43 (14.0%)	3/54 (5.6%)
Mepolizumab	7/367 (1.9%)	13/97 (13.4%) ***	3/248 (1.2%)	4/119 (3.4%)	3/43 (7.0%)	10/54 (18.5%) ^### §§^

ICS: inhaled corticosteroids; BDP-HFA: beclomethasone dipropionate HFA; LABA: long-acting beta-agonists; LAMA: long-acting muscarinic antagonists; OCS: oral corticosteroids; * *p* < 0.05, *** *p* < 0.001 vs. nFE; ^#^ *p* < 0.05, ^##^ *p* < 0.01, ^###^ *p* < 0.001 vs. NE; ^§^ *p* < 0.05, ^§§^ *p* < 0.01, ^§§§^ *p* < 0.001 vs. OE; ^†^ *p* < 0.05 vs. mFE.

## Supplementary Materials

The following are available online at https://www.mdpi.com/2077-0383/9/7/2226/s1, Figure S1: Blood eosinophils and blood neutrophils in the asthmatic population, Table S1: Other functional parameters in the asthmatic population.
