# Supplementary material for: Clinical Characterization of the Frequent Exacerbator Phenotype in Asthma
Source: J Clin Med. 2020 Jul 14;9(7):2226. doi: 10.3390/jcm9072226 (PMC7408982; doi:10.3390/jcm9072226)
Supplement: Supplementary file 1 [file jcm-09-02226-s001.pdf]

Supplementary Materials

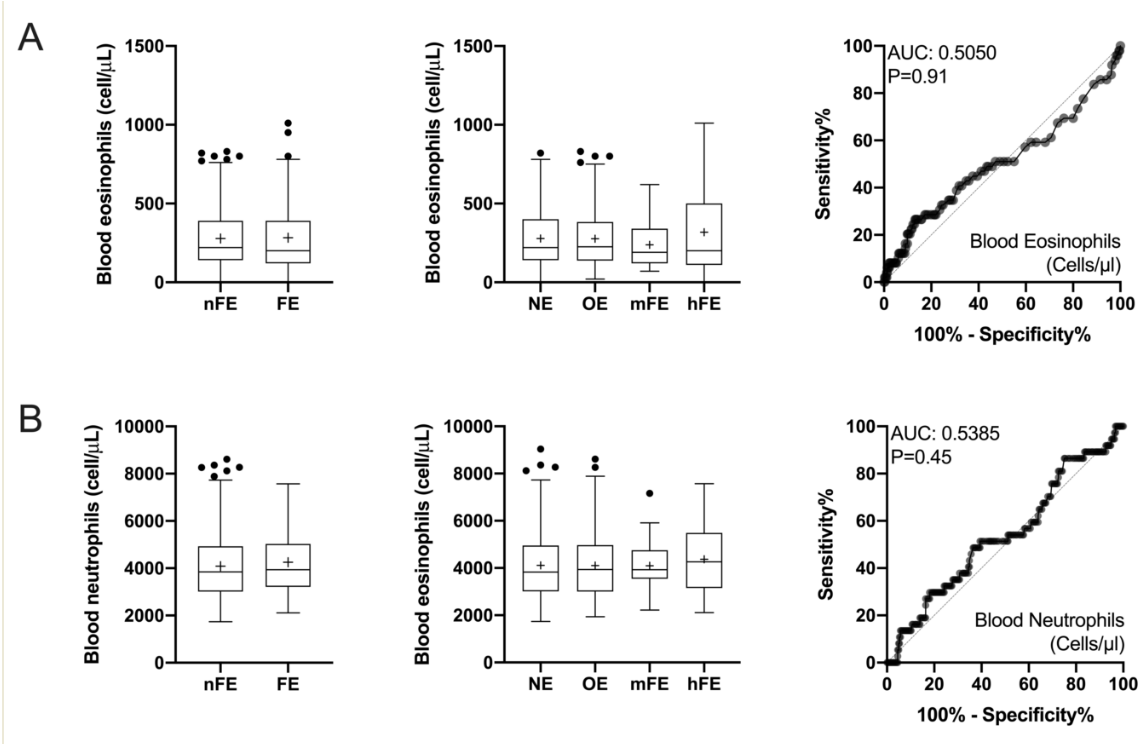

Figure S1. Blood eosinophils and blood neutrophils in the asthmatic population. Counts of eosinophils (A) and neutrophils (B) in blood and their respective curve analyses in which the hFE group was compared to all the other categories combined. Box-plots represent data from the first to the third quartile, whiskers represent the minimum and maximum value; "+" indicates the mean value.

Table S1. Other functional parameters in the asthmatic population.

**Functional Parameters**	**nFE (n=367)**	**FE (n=97)**	**NE (n=248)**	**OE (n=119)**	**mFE (n=43)**	**hFE (n=54)**
ΔFVC (mL)	220.4±332.4	235.3±274.5	201.2±236.6	265.4±483.0	168.6±275.0	289.6±266.8
ΔFEV_1 (mL)	243.5±217.7	205.1±184.8	246.5±185.2	237.6±271.7	186.4±148.4	223.8±215.6
FEV_1/FVC ratio (%)	69.1±12.6	66.6±10.7	69.5±13.3	68.3±11.1	67.8±10.9	65.7±10.5
TLC (%)	106.0±15.1	108.9±16.0	106.8±15.0	104.6±15.3	107.9±15.4	109.9±16.7
Heart Rate (bpm)	76±11	77±11	76±11	76±12	76±12	77±11
